# Book Reviews

**Published:** 1819-12

**Authors:** 


### CRITICAL ANALYSIS

OF

### RECENT PUBLICATIONS, IN THE DIFFERENT BRANCHES OF MEDICINE AND SURGERY;

### SELECT MEMOIRS, AND HISTORIES OF CASES;

### In the Literature of Foreign Nations.

Πατρίδος ἆρα
Ανδράσιν, ὦ πατρῷς ἄνδρες, ἀγαλλόμεθα.

***Discourses on the Elements of Therapeutics and Materia Medica.*** By NICHOLAS CHAPMAN, M.D. Professor of the Institutes and Practice of Physic and Clinical Practice in the University of Pennsylvania, &c. 2 vols. 8vo. 1817 and 1819.

[*Concluded from p.* 430.]

IN fever, when the intestinal canal is not primarily affected, and when the functions of the liver are deranged, Dr. Chapman judiciously advocates the use of purgative remedies; but he slightingly notices the practice of treating typhous fever by purgatives solely, which has of late been "much the vogue." In most of the cutaneous diseases of an inflammatory character, he advocates the use of laxatives: he has pursued the purgative plan invariably in scarlatina, as it ordinarily occurs in the United States, with the occasional use of the lancet; and he has "always had abundant reason to be satisfied with its efficacy. It has appeared to be calculated, not only to cure the disease in a more summary manner than any other mode of treatment, but likewise to afford the best means of preventing dropsical swellings and other derangements of health, or of removing them, when, by negligence or unskilfulness, they have been permitted to take place."

In some forms of acute rheumatism of long duration, when venesection fails to afford relief, even "augmenting the excitability of the vessels, and thereby aggravating the mischief," Dr. Chapman has found that active purging will occasionally prove of great advantage. "It seems, more than any other remedy, to quiet the mobility of the arteries, and to diffuse the excitement over the system, which, in this case, is chiefly concentrated in the blood-vessels."

After noticing the connexion often observed between rheumatism and cholera, dysentery, and other affections of the bowels, the author continues to remark, "Nothing, indeed, is more common in the practice of physic, than to see rheumatism suspended, or even cured, by diarrhœa, spontaneously induced. Taught by this fact, the course which nature points out, I have often imitated it, in the treatment of the more obstinate and protracted cases of the disease before us, and have had much reason to be satisfied with the results."

Modern practitioners, in general, are too little disposed to attend to indications of this kind; or, if they do attend to them, it is without considering the periods of diseases at which these spontaneous salutary revulsions take place;—a point of the utmost importance, and so carefully studied by the ancients.

The results of accurate observance and judicious reflection may be usefully adduced, although they may not be novel, especially when they tend to complete the destruction of deeply-rooted prejudices: we therefore transcribe Dr. Chapman's remarks on the use of this remedy in gout. "I have now," he says, "for several years, habitually employed purgatives in the paroxysms of gout, and with equivocal advantage. Not content with simply opening the bowels, I completely evacuate, by active purgings, the entire alimentary canal. This being accomplished, all the distressing sensations of the stomach which I have mentioned, are removed, the pain and inflammation of the limb gradually subside, and the paroxysm, thus broken, speedily passes away. To effect these purposes, however, it is often necessary to recur to the remedy repeatedly."

We have ourselves sometimes observed, that the pain has been alleviated by this mode of treatment, though the swelling and inflammation has continued apparently stationary for some time afterwards, results exactly similar to what ensue from the *appropriate* use of cold topical applications.* On this subject we witness, in a particular manner, the neglect, and even reprobation, of a useful remedy, because the views of the proposer of it have not been correctly discerned.

On the subjects of apoplexy, palsy, hydrocephalus, and mania, the author evinces an intimate acquaintance with the latest observations published on this side of the Atlantic; and adds his testimony to that of our best authors in favour of the use of purgatives in those diseases. In palsy, especially, he has carried it to a considerable extent, and with the most happy results: but here, as well as in mania, it is often not only necessary, Dr. Chapman says, to excite catharsis, but to give "large doses of drastic cathartics, so as violently to gripe, and otherwise harass and torment, the bowels." It is by this intense counter-irritation that, in the more severe cases of mania, the morbid action must be diverted from the head; and it is probably thus that the spirit of turpentine acts so beneficially in many cases of epileptic insanity. The same views regulate the treatment of chorea. Dr. Chapman thinks there is scarcely any chronic affection of long standing that yields more readily to any plan of treatment, than chorea does to purgatives; but, in order to effect this, "the impression once made on the bowels is never to be permitted wholly to subside. Without this is done, relapses are apt to take place, and we lose all which we had previously gained."

Epilepsy, the author considers to arise very frequently from disorder of the alimentary canal; and here, too, "cathartics must be re-

* See the very ingenious dissertations on Sensation, by Mr. SMITH of Bristol, in the xxvth and xxvith volumes of this Journal, for some highly interesting illustrations of this subject.

peated day after day, without interruption, unless imperiously forbid by peculiar circumstances. By continuing this course for several months successively, I have cured several cases of the disease completely, and afforded considerable relief in some others." The author, of course, employs auxiliary measures, according to the indications of the symptoms.

So many instances of the good effects of purgatives in the treatment of tetanus have been lately published, that we need not dwell on this subject any longer than to say, that Dr. Chapman's observations, as well as his reasoning, have pointed out their utility in that affection. He makes some remarks on the too indiscriminate use of drastic cathartics in dropsy: "Every practitioner," he says, "has seen it associated with fever, and no inconsiderable degree of even inflammatory action, [an origin of the disease by no means of recent discovery, since it was distinctly noticed by Stahl.] Exactly as the case assumes this aspect, does it indicate the use of the *lancet*, the saline purgatives, and especially the soluble, or cremor, tartar. But, in the selection of cathartics, it is much more common to prefer the drastic species, or what were formerly called hydragogues. Medicines of this character are indisputably mischievous under the circumstances mentioned, and are only advisable where the alimentary canal is torpid, the habit generally phlegmatic, without fever or local visceral disease."

In their application to hysteria and chlorosis, Dr. Chapman, as on many points already noticed, coincides in opinion with the author of the work on Purgatives; but, though there is this similarity of sentiments, it is not from Dr. Chapman having borrowed from Dr. Hamilton, as might be supposed; since, nearly twenty years ago, he read a paper before the Medical Society of Philadelphia, in which his doctrine of the gastric origin of those and other diseases to which we have alluded, and the propriety of the use of purgatives in their treatment, was distinctly advanced.

Many of Dr. Chapman's most valuable observations and reflections are dispersed in his discourses on the application of the particular species of each class of remedies: this obliges us to adduce some detached passages from these, although we cannot give a regular analysis of the whole. The following remarks, although made with so much earnestness by HIPPOCRATES, and appreciated by all good observers, are not generally acknowledged:

"It is a very curious fact, but one fully confirmed by experience, that, urged to any extent, evacuations from the bowels are found, in the complaints of the lungs, always mischievous; and, in some cases, so injurious as to be wholly inadmissible. Even in pleurisy, we cannot purge with the same freedom as in other cases of acute inflammation. But, in the chronic pneumonic affections, and especially in pulmonary consumption, the system immediately sinks under the operation of purgatives; and hence we are so careful to restrain diarrhœa in this disease."

Dr. Chapman speaks in very favourable terms of the use of olive-oil as a laxative. It is commonly employed in France, especially during pregnancy; and is certainly a mild, agreeable, and efficacious medicine on these occasions. Many old nurses in this country recommend it for the same purpose; but it is, we believe, bu rarely prescribed by medical practitioners.

Charcoal is another medicine, respecting which the author thinks more favourably than most other physicians; as a laxative, as well as to correct the disordered state of the contents of the intestinal canal, "in acidity of the stomach, in pyrosis, and in some of the stages of dysentery, when the stools are acrid and highly offensive."

Dr. Chapman speaks of the *black hellebore* as the species employed by the Greeks in mania; but here he is not correct: various passages in Pliny, and in Aulus Gellius, show that it was the *white* species they employed in the treatment of that disease.

We shall wholly pass over the discourses on *diuretics* and *diaphoretics,* although they contain many dispersed observations possessing some originality; but we have not much more space to allot to this review, and this must be principally given to the dissertations on the *diffusible stimulants.* We premise a few extracts from those on *expectorants* and on *emmenagogues.* The following aphorism respecting the use of the former, is consonant with some remarks already adduced: we need not apologize for transcribing it.

"Carefully avoid purging, as remarked on a former occasion, none of the complaints of the lungs will bear this evacuation to any extent. Besides which, the action of the secretory vessels of the lungs and intestines, would seem to be alternate and opposed. Expectoration, at least, is uniformly suppressed or diminished by diarrhœa or by purging."

The decoction of the inner bark of the red elm, (*ulmus* of Linnæus, Tournefort, and Jussieu; *ulmus rubra*, of Mecklenburgh;) has been recommended in this country in dysenteric affections; but, we believe, its efficacy has never had a fair trial. Dr. Chapman says, "It is known to many, that the late Dr. Grant, of Virginia, had for nearly half a century an unrivalled reputation in the part of the country where he resided, in the management of dysentery. As he once informed me, his practice consisted in little more than purging moderately in the commencement of the case, and subsequently pouring in very freely the elm mucilage. By this alone, he declared, that the bloody stools, tormina, tenesmus, &c. were more speedily removed than by the ordinary remedies. Even admitting that one-half of this statement is correct, the article will still appear to be strongly deserving of attention."

Dr. Chapman, as we stated on a former occasion, considers the menstrual fluid to be a *secretion;* but, although his physiological reflections on the nature and apparent causes of the functions producing it are in general judicious, his arguments in favour of a secretory action of the vessels of the uterus, seem to us to indicate no more than what occurs in all the *exhalations;*—a distinction made by Bichat, that is not, it seems, generally understood.

The *polygala senega* was discovered by Dr. Hartshorne of Philadelphia, the author says, to possess admirable properties as an emmenagogue: Dr. Chapman has given it a trial "to a considerable extent, and with sufficient success to warrant me in recommending it as one of the most active, certain, and valuable, of the emmenagogues." Next to this, he seems to place the *juniperus sabina.* The latter we know, from observation, to be a valuable remedy in many cases to which it is not commonly applied; but it requires so much caution in its use, and, if any thing like inflammatory action be present in persons of a vigorous habit, is so hazardous a remedy, that it is not probable it will ever be extensively employed.

The savine seems to act by exciting especially the sensibility of the uterus; and, since there are many cases of amenorrhœa in which this is really the proper indication for the treatment, we cannot too far extend our knowledge of substances apparently possessing the same power. Dr. Granville, some years since, in a paper inserted in this Journal, spoke in favourable terms of the efficacy of madder in that respect; but it has not, we believe, received a full trial, perhaps from Cullen having expressed so unfavourable an opinion respecting its use. Dr. Barton, of Philadelphia, is disposed to think it efficacious: he says, "I have so frequently observed it to show considerable effects upon the uterus, that it is without any degree of hesitation that I speak of it as a remedy worthy of the attention of practitioners. It appears to me to be suited both to cases of retention and suppression of the menses, though it is chiefly in the latter condition that I have employed it."

After some remarks on the cases in which it is advisable to depress action, for the removal of this affection, and on the treatment of dysmenorrhœa, Dr. Chapman says, "After all, however, we are not to lose sight of the use of mercury in this disease (amenorrhœa): by a moderate salivation, continued for a week or two, we shall sometimes succeed in curing it, in its different forms, when every other mode of treatment has failed."

Dr. Chapman commences his discourse on the *diffusible stimulants*, by observing, that, "in relation to general stimulants, there is a distinction too important to be overlooked. As formerly remarked, we have a set distinguished by great diffusibility, and which, nearly as soon as exhibited, occasion universal excitement over the body; and there is a second section, by which tone is imparted, though very slowly, and only by a long-continued administration. The diffusible are very transient in their effects; while such as are more gradual in their operation, produce permanent or enduring impressions, and are called tonics." The division thus marked out is, then, that adopted by the author. We have already expressed our opinions respecting its propriety, and have shown that Dr. Chapman is aware that it does not rest on a solid and physiological foundation.

"In discussing the properties of the first class," he adds, "it has been usual of late to arrange the articles under the two heads of narcotics and antispasmodics. But the latter term is an exceedingly vague one, and to which it is not easy to attach any definite idea." From the opposite states of the system in which spasms occur, he shows that, in one view, we should treat, under the head of antispasmodics, of all the stimulating and tonic remedies; and, in another, of those that are directly evacuant and depletory.

After some remarks on the controversy carried on, especially during the latter part of the last century, respecting the immediate effects of the narcotic medicines, he advances his own opinions, which do not bear the character of precision generally evident in his reasonings; for, although he considers that the immediate agency of those substances is really excitant in certain doses, he says, "by a large dose, debility, without any previous excitement, is occasioned; or, if there be excitement, it is so transient and evanescent as not to be perceived; and, when the effect goes off, there are tremors, sickness, head-ach, and oppression. Taken in excess, the system at once sinks under the impression, and the consequences are either wild delirium, or a heavy stupor, deep, difficult, and stertorous respiration, convulsions, apoplexy, or paralysis, and ultimately death."

It is quite inconceivable how a substance, which is stimulant in a moderate dose, can be immediately sedative in a larger one. But, perhaps, Dr. Chapman does not mean to signify that it is locally, but generally, so. This may be readily thus explained: the larger dose, by producing an intense concentration of the vital powers in the part to which the substance is applied, deprives the rest of the system of nearly or entirely all its vital powers; the consequence is immediate debility. Thus, a gall-stone, on passing the biliary duct, (which must, of course, be considered a local stimulant,) will produce immediately coldness of the whole surface of the body, dimness of sight, almost total loss of voluntary power, tremors, and finally syncope, or even instant death, without any organic lesion; an instance of which has recently occurred to our knowledge. An excessive dose of alcohol has been known to produce similar effects. It must be observed, too, that excessive afflux of blood to the brain, by causing compression of that organ, will produce symptoms similar to those considered to be signs of the immediately sedative agency of the narcotic substances.

As we have not yet given a specimen of Dr. Chapman's aphorisms respecting the use of particular remedies, many of which are really excellent, and some of them oracular, we shall do so on the present occasion; as it will save us the trouble of making our own abstract of his precepts for the use of the class of medicines under immediate consideration.

"1. It will generally be found best to begin with small doses, though we are to recollect that the action of these medicines, by repetition, is more rapidly lessened than any other; so that, in a short time, the doses require to be considerably augmented. Exceptions, however, exist to the precept which I have inculcated. Cases of typhous fever, and some of the neuroses especially, are marked by such a loss of susceptibility to impression, that we are called upon, even in the early stages of these disorders, to exhibit stimulants freely.

"2. It is wrong to combine many of these articles in one prescription, or to use any number of them at the same time. By directing them separately, or nearly so, we economize our resources in protracted diseases; and, probably, also make a more distinct and powerful impression. Where it is expedient to deviate from this course, we should be careful to select such articles as are calculated to co-operate to the same end. Much is sometimes gained by harmony in the action of medicines.

"3. It is advisable to change occasionally the part of the body to which we apply stimulants; as sensibility will be left in one place to a medicine, when completely exhausted in another. This is a principle of very extensive application in the practice of our profession. The excitability of the stomach being worn out, we should make an application to the bowels or skin. We have the propriety of this practice exemplified in the use of opium. Numerous are the cases when, after it cannot be any longer given with advantage by the stomach, it will act very efficaciously, if injected into the rectum.

"4. In the administration of stimuli, (as indeed of all medicines, but more especially stimuli,) we should endeavour to graduate the article to the state of the excitability. This is a point of infinitely greater importance than is commonly imagined. Between certain conditions of the system and certain medicines there would seem to be an affinity or relation, which, when consulted, often leads to decisive advantages. It is not always that the most active article produces the greatest effects. Castor-oil will sometimes purge, when a drastic cathartic proves wholly inert. In the low states of disease, I have witnessed, in some instances, more effects from wine-whey than strong toddy. This proceeds from the article being in unison with the condition of the system."

We shall select two subjects from the discourses on *the practical application of stimulants*, the *carbonate of ammonia*, and *opium*, for a partial analysis; since the consideration of the whole would extend this article much farther than can be permitted.

In typhous fever, after the increased or inflammatory actions have subsided, and when much and increasing debility comes on, Dr. Chapman thinks very favourably of the powers of the carbonate of ammonia. Cinchona, on those occasions, especially when the stomach is particularly inactive, we have often found to be productive of no benefit; and it frequently produces nausea and diarrhœa. Wine, too, sometimes affects the brain in an unpleasant way, causing delirium: here the remedy Dr. Chapman advises is of remarkable utility, and cannot, we think, be superseded by any other with which we are acquainted. Great caution is, however, required in its use, especially when there has been gastric inflammation. The muriatic acid is the medicine most generally resorted to on those occasions, by a physician in London, of extensive practice in typhous fever; and it is, perhaps, in general, the most safe and appropriate remedy.

"In districts subject to intermittent fevers," the author observes, "a species of pleurisy often prevails, which, seizing on the emaciated frames of the wretched inhabitants of such situations, is comparatively a feeble state of disease. Bleeding here, more than once, cannot be borne; and, soon after, the volatile alkali, and other stimulants, become necessary. The pneumonia of persons advanced in life, or of exceedingly delicate and debilitated constitutions, affords a second example where our medicine may be early employed. To deplete to any extent, under such circumstances, would be fatal; and sometimes, even from the commencement, we are compelled to resort to stimulants, among which no one is so proper as the carbonate of ammonia with opium."

In the winter epidemic of the United States, the *pneumonia typhoides*, it has been employed by the author with remarkable benefit.

Dr. Chapman notices the reports that have been made of the use of this medicine on the continent of Europe, especially in France, in apoplexy and palsy; but he says, "I confess I do not repose much confidence in these representations." Theory must here be silent. Experience has most clearly shown, that, by local bleeding after certain intervals, and the use of the carbonate of ammonia during the intermediate periods, many cases of paralysis have been benefitted in a very remarkable and peculiar manner. We are disposed to add our testimony in favour of these reports, having seen several cases of old persons of spare habit of body, who had suffered repeated attacks of paralysis, (hemiplegia in most instances, and generally with affection of the power of speech,) in quick succession, from which they had partially recovered; where local and general bleeding, regular and constant purging, blisters, &c. had not been of any apparent benefit; who, if not entirely relieved, have been preserved from fresh attacks, for several months, and even years, by being put on this plan of treatment. The increasing frequency of the attacks immediately previous to the adoption of this practice, would hardly permit a doubt of its efficacy. We have never had the means of ascertaining the nature of those cases by dissection; but we suspect, from the symptoms, that the fit depended on serous effusion in the cranium.

Dr. Chapman however says, "of palsy there is one variety, in which the carbonate of ammonia is unquestionably useful. It is the offspring of rheumatism. Being long affected by this disease, the muscles lose the power of contraction, and the extremities, if they be the seat of the complaint, of motion. Cases of this description have repeatedly come under my care, which so nearly resembled general palsy, [the history of the disease, we think, would in all cases clearly mark the distinction,] as not easily to be discriminated, though they may be generally known by more or less of pain or uneasiness, and particularly during damp and cloudy weather."

In the vomiting affecting drunkards and pregnant women, it is recommended by Dr. Chapman as of utility; and he particularly points out the advantage of choosing this, when stimulating remedies are resorted to in mixed or doubtful cases: that is, where inflammation is, or has recently been, present. Its effects are so evanescent, that, if given with caution, it can hardly be productive of much injury. We conclude this subject with the following passage; as Dr. Chapman is very far from being a *Brunonian*, it cannot be considered unworthy of serious attention.

"Not long before his death, the late Dr. Kuhn, who was one of the most sagacious and discriminating practitioners of this country, told me, (says the author,) with some emphasis of manner, that, after an experience of nearly half a century, if he were called upon to say with what single remedy he had done most good, he would without hesitation name the volatile alkali, aided by wine-whey. After such praise from such authority, it surely would be superfluous to press this article on medical attention."

Opium the author considers so direct and powerful a stimulant, that "it would not be difficult to run the parallel to a considerable extent between it and wine." Almost every practitioner must have met with persons, to whom it is more exhilirating than wine; but in those persons, the consequent languor is proportionably great. Yet, Dr. Chapman observes, wine and opium can hardly be substituted for each other, their effects are so different. Perhaps this arises from the one affecting chiefly the nervous system, and the other the fibrous structure. However, it must not be forgotten, that, though a stimulant, Dr. Chapman says, that property "is so tempered by its power of assuaging pain and doing away irritation, that in many instances its effects are different; and hence it may be safely and efficaciously employed where, proceeding on common principles, it would be prohibited."

The author adds his testimony to the favourable opinions that have been entertained respecting the use of opium in intermittent fevers, in the cold stage especially; and in the hot stage even, in conformity with Lind, in cases where the patients are of a spare and asthenic habit. The following sentiments are bold; but, with a little modification, they may be of useful application.

"In typhoid pneumonia, whether original or induced by improper management of the inflammatory form of the disease, our medicine is indisputably one of the most decisively useful remedies. On this point no difference of opinion exists; and we may even go so far as to lay it down as a principle, that, in all the varieties or stages of pneumonia, where venesection is forbid or is an equivocal measure, opium should be employed; uniting with it small portions of ipecacuanha and calomel, or bringing into co-operation the volatile alkali, as the one or the other may seem to be preferable."

A physician of considerable reputation, now practising at Edinburgh, is said to treat nearly all cases of pneumonia with opium, in large doses, and to be remarkably successful in his practice. We think it proper to adduce these remarks; but we by no means advise the use of what we consider to be a very hazardous measure. The whole weight of the arguments in favour of the application of opium just alluded to, as well as some ingenious suggestions respecting the mode of using it, will be found in the treatise of Mr. Ward of Manchester.

Dr. Chapman terminates his remarks on the use of opium in gout, by noticing the cause of the death of Brown; he could hardly have concluded them in a better manner. In tetanus, he is not inclined to think very favourably of it; yet, he says, "it should be recollected that the few instances of tetanus which are reported to have been cured by opium, were by very unusually large doses." It should also be recollected, that, of the recorded cases which have terminated favourably in England during the last twenty years, opium was exhibited in by far the greater proportion of them. In our opinion it is, if not the most efficacious remedy, distinctly considered, at least a very powerful auxiliary to blood-letting and purgatives. But Dr. Chapman is not disposed to think favourably of opium in any diseases of the class *neuroses:* he thinks these are most successfully managed by evacuations of the alimentary canal, and by venesection. When treating of the *mania à potu*, or, as Dr. Sutton terms it, *delirium tremens*, Dr. Chapman says, "the opiate treatment, claimed within the last few years, by several of the European writers, as a great practical improvement, has been known, and generally adopted in this city, as far back as recollection or traditional reports extend." We seem to owe the introduction of the practice to Dr. Young of Newcastle.

In *puerperal mania*, the author is not very warm in the praise of opium: he, however, thinks the disease "to be more frequently attended with extreme *nervous irritation* than *inflammatory* action. In the former state, I have seen the most manifest advantage from large and repeated doses of the tincture of hop, or the camphorated emulsion, where opium unequivocally aggravated the symptoms."

We shall adduce one or two more observations from this work, and then we must conclude. Dr. Chapman gives his testimony in the most unequivocal manner in favour of the use of *spider's web* in ague, so forcibly inculcated by Dr. Robert Jackson.* He says, "The spider's web has been for some time past pretty liberally prescribed by myself and several of my medical friends, and particularly by Dr. Physick and Dr. Dewees; and, though we attach different degrees of value to the article, are all satisfied that the representation of its virtues to which I have referred, (that of Dr. Jackson, in his work on Fever,) is very little, if at all, exaggerated."

The author's discourse on *mercury* is a highly valuable part of the work; not that it contains much that is novel, but from the methodic and comprehensive manner in which the subject is treated, and the judicious pathological and practical remarks with which it abounds. In regard to *venereal diseases*, Dr. Chapman nearly coincides in opinion with Mr. Carmichael; though this seems to have arisen from original observation, not an adoption of the sentiments of that accurate observer.

If we have given any thing like a correct idea of the character of Dr. Chapman's Discourses, have furnished the reader with some useful hints, or impressed on his mind some good practical precepts, we have effected all that we attempted. It must be obvious that a regular and complete analysis of a work treating of the particular application of all the materia medica in general use, and comprising pathological observations and reflections on nearly all the diseases incident to the human body, could not be comprised in such an extent of space as is appropriate to the limits of our Journal. There is, however, one considerable fault in the work which we must point out, and which

* See *London Medical and Physical Journal*, vol. xxi. 353; xxii. 369.

has arisen from its bearing its original form of lectures. The author has but rarely referred to their sources the opinions he has adopted from others, and when he has done this, it has been merely nominally, without any express allusion to the writings in which they may be found: hence, those students who may desire to contemplate more fully the observations and opinions thus noticed, will have great difficulty in finding them; and, from many of them having been published in Journals, his enquiries will not often be attended with success. Some additional notes, the making of which would not have been attended with much trouble to the author, would have supplied the deficiency.

---

### *An Account of the Structure of the Brain and its Appendages.* By Professor Lauth.

[*Continued from page* 339.]

§ I. Of the cerebrum. 3°. *The department of the crura.*—From the floor of the two lateral venticles, there arise the tubercles called the corpora striata and thalami of the optic nerves; which form together the superior portion of the organs known by the name of the *crura cerebri,* at the base of which these are situated. These organs, the tænia semicircularis, the third ventricle, with the anterior and posterior commissures, constitute the *department of the crura cerebri.*

A. *The corpora striata.*—The corpora striata are two large tubercles, placed at the anterior and exterior part of each ventricle, where they form two convex masses, large and spherical at their anterior extremity. Each of them becomes narrower as it passes backwards along the exterior margin of the thalamus opticus, and terminates in a point. They are composed of grey substance at their unconnected surface, but present inwardly stria of white and cineritious matter.

B. *The tænia semicircularis.*—At the internal margin of each corpus striatum, a band of about a line in diameter is seen, which has its origin in the anterior lobes of the cerebrum, passes at the side of the anterior pillar of the fornix, and is continued between the corpus striatum and the thalamus opticus, and descends under the inferior horn of the lateral ventricle, where it disappears. Vicq-d'Azyr* called this band *fibrous,* because fibres are seen in the thin membrane which covers its surface. Wenzel† attributes its colour, (yellowish,) to a sort of lymph effused beneath this membrane.

C. *The thalami optici.*—The thalamus opticus is the second tubercle, situate in the lateral ventricle, within and behind the corpus striatum, at the interior margin of the tænia semicircularis; its surface is convex, extensive and spherical anteriorly; it becomes more contracted posteriorly and externally; its exterior margin is curved, the interior edge is rectilinear and turned towards the correspondent margin of the other thalamus; its substance is white outwardly, and becomes grey interiorly.

A tubercle of a smaller size is placed on the surface of each thalamus.

Between the interior margin of these two eminences, we find a grey substance, noticed by Haller, Sabatier, and Soemmering, and termed

---

* *Traite d'Anat. et de Physiol.* pl. v. vi. viii. ix.

† *De Penit. Struct. Cereb.* p. 85.

the *soft commissure* by Vicq-d'Azr.* Wenzel† has found it larger in brute animals than in man, whence it is that the third ventricle in them is proportionably smaller.

The posterior and more contracted part of the thalamus opticus, descends with the inferior horn of the lateral ventricle, where it is divided, as Reil remarks, into two productions, one of which unites with the tænia semicircularis, and to the descending portion of the corpus callosum; the other goes down to the basis of the cerebrum, where it surrounds the crus cerebri, and is continuous with the optic nerve.

D. *The third ventricle.*—The corpora striata and the thalami optici are united towards the superior border of their internal margin, by means of the soft commissure above mentioned. When they are separated, we perceive the *third ventricle,* a canal extending from before backwards, below the fornix, and beneath the lateral ventricles, with which it communicates by means of the foramen in the septum lucidum. At each extremity of the third ventricle there is a hole; the anterior, called the *vulva,* leads to the *infundibulum;* and the posterior, termed the *anus,* is the commencement of the *aqueduct of Sylvius.*

E. *The commissures of the cerebrum.*—Above each hole in the third ventricle, there is a medullary cord, the fibres of which have a transverse direction. These two cords are called the *anterior* and *posterior commissures* of the cerebrum. They serve to establish a communication between the two lateral masses of this organ.

The anterior commissure, placed before the anterior pillars of the fornix, continues its course on each side, so as to form a sort of arcade, the convexity of which is direct anteriorly; and it terminates with a striated appearance in the anterior lobe of the cerebrum. On examining a hardened brain, Reil has found it like a nervous cord, which might be separated from the mass of this organ, in the interior of which there remains a canal after the cord has been drawn from it.

The posterior commissure, which is prolonged in the same manner in the cerebral mass, is curved inwardly without forming a white cord so decisively marked as the former.

F. *The crura cerebri.*—We have termed the corpora striata and the thalami optici the superior portion of the crura of the cerebrum; it is evident that they really form it when we examine the structure of these tubercles.

If we remove, by scraping it, the substance of the corpus striatum and the thalamus opticus, we find the interior of the former present a radiated appearance of white and grey substance, and that the interior of the thalamus opticus presents a grey cineritious appearance. When we arrive at the base of the two tubercles, a sort of packet presents itself, the central portion of which is grey; and this is surrounded by a white band, which another striated band of white and grey matter again envelops. This packet, composed of three portions, is bordered by a large band, of grey and white substance, arranged in rays directed towards the circumference of the anterior and middle lobes. After having traversed a certain extent, these rays end in the white and homogenous substance, terminated by the convolutions of the cerebrum.

* *Traite d'Anat. et de Phys.* pl. f. 1.

† *De Penit. Struct. Cereb.* p. 129.

Thus, the corpus striatum and the thalamus opticus are a nucleus from which arise radiations extended towards the hemisphere; or, reciprocally, the convolutions of the cerebrum, and the cerebral mass, at first white, and then radiated, are concentrated in the corpus striatum and the thalamus opticus.

If we now reverse the encephalon, we perceive, at its basis, the *crura cerebri*, or two medullary trunks coming from each hemisphere, in a direction from without inwardly, and from before backwardly. Each crus is encircled by the optic nerve, a large medullary cord, which descends from the lateral ventricle in a direction from behind forwardly. The crura are united, at an acute angle, towards the median line, to give origin to the pons of Varolius, of which they form the anterior and superior portion. Their inferior surface is white, and marked by longitudinal stria; when we scrape them, we find their substance become grey, and perceive in their interior a crescent of black or deep grey matter. We afterwards find, on continuing to scrape in the direction of the crura, white and grey stria, which Vicq-d'Azyr* termed a corded layer of white substance in the thalami optici, through which we discern the grey substance of the corpus striatum, which has received from Reil† the name of the radiated circle. We come then, proceeding from below upwards, to the corpus striatum and the thalamus opticus, which form the superior portion of the crura. We may consider these as the concentrated hemispheres, or rather, the hemispheres are an expansion of the crura. We shall, however, perceive, on taking a view of the plates of Vieussens,‡ that the corded layer of Vicq-d'Azyr, and the radiated circle of Reil, are not a new discovery, but that the honour of having first discovered them is due to the physician of Montpelier, whose observations have only been developed in a more precise manner.

4°. *The appendix of the cerebrum.*—The cerebrum, at liberty in the osseous vase which encloses it, is connected by the *infundibulum*, to a gland enveloped in a fold of the dura mater; it is in this manner fixed to the cranium.

A. *The pituitary gland.*—The pituitary gland is a spherical body, lodged in the sella turcica, and placed between the laminæ of the dura mater, with the exception of its superior part, by which it communicates with the infundibulum. This body, of a reddish-grey colour, vascular, and tubulated, is composed of a peculiar substance, and was called a gland by the ancients, who believed its office to be, to transmit the pituita of the brain into the nasal cavities.

The moderns have advanced a multitude of opinions respecting this gland. Malacarne§ found it to vary much in its situation, its volume, and its lobes. Gall|| gives to it, as well as to the pineal gland, the appellation of a ganglion enveloping nervous filaments. It is considered by Carus,¶ as the superior central ganglion of the ganglionic system: he found it** in the fœtus, very large, and similar to the brain. Wen-

* *Trait. d'Anat. et de Physiol.* pl. xii. xiii. xiv. xxi. xxii.

† *Archiv. für die Physiologie*, ix. 147, 259; tab. ix.

‡ *Neurographia*, c. 13; tab. xvi. B.

§ *Encefalotomia*, ii. No. 114.

|| *Anat. et Physiol. du Cerveau*, i. 316.

¶ *Darstellung des Nervensystems*, p. 254, 269. ** *Ibid.* p. 282.

rEIL* divides it into two lobes, one small and anterior, the other large and posterior, and pretends that those two lobes are united by a membrane; he distinguishes in the great lobe, an external and red, and an internal and white, substance; he remarks in the small one, two little fossæ; one to the left, the other to the right, from each of which arises a little canal which goes to the posterior margin, by which the infundibulum is attached to it. He admits a relation between the pituitary and pineal glands, placed at the two extremities of the third ventricle; and asserts, that no part of the brain diminishes so much in old age as this gland. In many diseases, especially in those caused by grief, it has appeared to him to be softened, and converted into a hardly consistent pulp: and he attributes the causes of epilepsy to a morbid state of this body, to which he gives the name of the *appendix of the cerebrum.*† I have also occasionally found the pituitary gland in a diseased state; but the bodies of the epileptic patients which I have examined, have not furnished me with any determinate facts respecting it; the gland having been always found in a perfectly natural state.

B. *The infundibulum.*—The infundibulum is a conic pedicle, situate at the base of the cerebrum, before the mamillary eminences, and attached by its larger extremity to the cerebrum from which it appears to arise. By its smaller extremity, it is attached to the pituitary gland: its substance is of a reddish-grey colour. It was believed to be traversed by a canal destined to conduct the serum of the third ventricle into the pituitary gland. Modern anatomists assert that it is solid, because it is not filled when mercury is thrown into the third ventricle. VIEUSSENS,‡ however, assigns to it a spongy structure, because he observed that coloured alcohol, passed into the third ventricle, was diffused in the pituitary gland. This opinion much resembles that which WENZEL§ has advanced, who saw the posterior lobe of the gland filled with mercury, after having introduced the syringe into the infundibulum, whilst the anterior lobe did not receive any of the metal. This author considers the pituitary gland as a secretory organ, which transmits its fluid, by the cellular substance of the infundibulum, to the third ventricle of the brain. According to this hypothesis, the commencement of the apparatus would be the gland, and the termination the infundibulum.

§ II. THE CEREBELLUM. The cerebellum is the posterior and inferior part of the mass of the encephalon. It is situate in the occipital fossæ, below the tentorium, which it is necessary to divide to discover it. The posterior lobes of the cerebrum, placed on the tentorium, above the cerebellum, are also extended more posteriorly than the latter. Its substance is both white and grey, and, like the cerebrum, the grey enters into the grooves, and envelops the convolutions of the white substance. The structure of this organ is more complex than that of the cerebrum, because of its lamellated disposition. It has been particularly well developed by MALACARNE‖ and REIL.¶ The cerebrum of the fœtus is

* *De Penit. Struct. Cereb.* p. 207, 236.

† *Beobachtungen übera den Hirnanhang,* 4to. Maine, 1810.

‡ *Neurographia,* c. 8.

§ *De Penit, Struct. Cereb.* p. 252.

‖ *Nuov. Esposizione della vera Struttura de Cerveletto Umano,* 8vo. Torino, 1776.

¶ *Archiv. für die Physiol.* viii. sq.

relatively less than the cerebrum, according to CARUS.* In vertebral animals, with the exception of the *Sepia*, ROLANDO† has found no difference between the cerebrum and cerebellum: whilst WENZEL‡ asserts that there is a greater than in man.

Externally, the cerebellum is divided into two *hemispheres*, united by an intermediate body, which is termed the *vermiform eminence*, (or process.) Each hemisphere presents two surfaces, the one *superior*, the other *inferior*. The middle of the latter presents along the vermiform eminence, a sort of groove, which is termed the *valley*, and is destined to lodge the medulla oblongata. The two surfaces are separated in each hemisphere by a *horizontal groove*. The superior surface is divided into two *lobes*, the one *anterior*, quadrilateral; the other *posterior*. There is, besides, on this surface, and towards the anterior extremity of the vermiform eminence, a common *central lobe*. The inferior surface presents three lobes, the *digastric*, the *soft*, and the *posterior*. Each lobe is subdivided into *lobules*, *folicles*,§ and *laminæ*.

The vermiform eminence is shorter than the hemispheres, whence arises a space between its anterior extremity and the latter, which is termed the *semilunar curvature;* as well as another space between its posterior extremity and the hemispheres, to which the name of the *saxiform curvature* has been given. According to its two surfaces, the eminence is itself divided into *superior* and inferior. The latter, placed in the middle of the valley, is parted out from before backwards in the following manner: the *transverse commissure;* the *transverse bands*, some of which are short and superficial, others long and deep-seated; the *pyramid;* the *plug*, and, lastly, the *uvula*, which forms the posterior extremity of the eminence.

There are some other parts still to be noticed in the valley. What are called the *amygdales*, are two globular bodies, situate between the concave surface of the digastric lobe, on one side, and the plug, the uvula, and the posterior valve, on the other side. The *floccules*‖ are two appendices, situate between the amygdales, the crus of the cerebellum, and the medulla oblongata. The two semicircular cavities, placed between the amygdales, the inferior transverse band, the plug, and the uvula, bear the name of the *swallows' nests*. The *posterior valve*, or *posterior veil*, is formed by two semilunar, medullary, laminæ, each of them running in a direction from the uvula towards the floccule. These laminæ were discovered by TARIN,¶ and called by him the *posterior*, *infe-*

* *Darstellung des Gehirns*, p. 283.

† *Saggio della vera Struttura del Cervelle*, p. 14.

‡ *De Penit. Struct. Cereb.* p. 17.

§ The introduction of this term requires an apology: there is no word in the English language that expresses the meaning of the original; *(feuillets*, little leaves,) a single word for it is necessary, or at least very desirable; the translator has therefore been induced to adopt that alluded to.

‖ *Little tufts* or *tassels*.—The apology for the word *folicles* will apply to this. The translator must not be blamed for the barbarous language contained in this and the former articles on the same subject; (the intermixture of pure Latin with English words is expressly alluded to;) he has not thought it allowable for him to substitute new terms for any that custom had established: the use of the word *groove* for *sulcus*, and some other trifling ones, must be excepted.

¶ *Adversaria Anat.* p. 8.

*rior, semilunar, valves.* Malacarne* also named them the *semilunar valves.*

Two modes have lately been pointed out for developing the interior structure of the cerebellum. The one, described by Reil,† consists in hardening it, and tearing it from the surface towards the centre, parting from the horizontal groove. In the other mode, we place, with Gall,‡ the point of the scalpel on the crus of the cerebrum, near the auditory nerve; we cut this, by a vertical stroke, in the direction of the crus, and we prolong the incision into the hemisphere, beyond the middle of which it should not pass, because the exterior two-thirds of it should remain undivided.

The *crus* of the cerebrum, is the medullary nucleus or trunk, which arises from the superior and interior portion of the hemisphere. It does not contain white matter solely; for, interiorly, and nearer the superior than the inferior surface, we find a grey substance, called the *rhomboid body; cineritious isle;* the *festooned, ciliary, dentated,* or *fringed, body.*§ This body is contained in a flattened capsule, from which it may be extracted; its form is triangular, and its summit directed forwardly. Each crus is divided into *branches, ramicles,* &c. generally designated by the term *arbor vitæ;* of which anatomists, the moderns especially, have not taken the trouble to give an exact description. These divisions may be thus displayed. As the surface of the cerebellum is composed of lobes, lobules, &c. so the crus is divided into branches, ramicles, &c.; and we perceive this construction, either on tearing it, according to Reil, by commencing at the horizontal groove; or by cutting it, according to the mode of Gall. On making these preparations, we see the two branches of the crus that terminate in the two lobes of the superior portion of the hemisphere, and the three branches which appertain to the inferior portion. The ramicles of each branch are directed towards their respective lobes, and their subdivisions towards the folicles and laminæ. Malacarne, who enters into the most extensive details on this subject, makes the quadrilateral lobe to be composed of five lobules: the first formed of three folicles, the superior of which encloses the laminæ, &c. Reil asserts, that there is a sort of articulation which consists in one lamina being engaged between two others. It is from this cause, that the mass of the white substance is not diminished by subdivision. Here then, as in all the other parts of the cerebral organ, this substance is formed in the same place that it occupies, and it is derived, like the blood, from one and the same source.

The cerebellum, of which we have described the interior structure, has important communications with the three central organs of the encephalic mass, the two pontes, and the medulla oblongata. These communications consist in three medullary branches of considerable size, which come from the division of each of the crura of the cerebellum. The *posterior branches, testicular processes of the cerebellum, rising portion of the crura of the cerebellum,* and *columns of the medullary valve,* proceed from below upwards to the posterior tubercula quadrigemina,

* *Cerveletto,* No. 81.

† *Archiv. für die Physiol.* viii. p. 290.

‡ *Anat. et Physiol. du Cerveau,* i. p. 256.

§ The common term for it in the London schools is the *locus niger.* Edit.

with which they unite by a white medullary cord: they are thin, roundish, and a little covered by floculent processes; the large valve is placed between them. The *lateral branches, middle portions*, or *third portions*, are the crura themselves, large and rounded, which traverse from the hemispheres to the pons of Varolius, of which they form an integral portion. The *anterior branches, spinal medullary processes of the cerebellum, peduncles*, or *columns of the medulla oblongata*, descend from the crus of the cerebellum, in the form of a thin, flat, ribbon, to terminate at the lateral margin of the medulla oblongata.

[*To be continued.*]

---

### *An Account of a Case of want of the Uterus, discovered on performing the Operation for imperforate Vagina; with some Remarks and corollary Observations.* By George William Stein, Ordinary Professor at Bonn.*

The history of this interesting case is related with much minuteness by Professor Stein, particularly those points of it respecting the operation; apparently, from some fear that blame might be attached to him for his conduct on the occasion, because he had not sooner discovered the nature of the malformation: this, however, is really groundless. We shall here give only an abstract of it, and of the corollary instances adduced by Professor Stein; omitting, however, nothing that we consider to be important, either in a physiological or medical point of view.

A lady, twenty-four years of age, had been married five years without having ever menstruated; although the orgasm generally accompanying the appearance of the menses, with some distress about the pubes, had recurred at the ordinary periods. After having expected the establishment of this function until her hopes were almost lost, and perceiving her health to be a little on the decline, being affected with oppression of respiration, and occasional fits of fainting, she permitted a midwife to examine her, for the purpose of ascertaining if there existed any mechanical obstruction. A firm membrane, supposed to be the *hymen* in a state of unusual thickness, was detected; Professor Stein was consequently consulted. He describes the patient as presenting the most perfect characteristics of the female form: slender and delicate; with full breasts, a fair complexion; and an animated countenance, expressive of the finest sensibility: her manners were particularly delicate; and she seemed, indeed, to be a model for her species. On making a slight examination in the erect posture of the patient, the account of the midwife appeared to be correct; and the usual instruments were prepared for dividing the membrane in the ordinary way: but, on making pressure on it with the finger and scalpel, it retreated inwards, about half the common depth of the vagina, and it was then discovered that this membrane formed a sort of sac. But, from the characteristic habit of the lady, and the maladies she had experienced, no suspicion of a want of the uterus was entertained: it was considered that the sac had been originally a plane membrane of the kind so commonly met with in the vagina of females, which

---

* *Ein höchst Seltener Fall eines gänzlichen Mangels des Uterus. Endeckt bei versuchter operation einer vermeintleih gewöknlichen Atresie und zur Warnung bei ähnlichem Verhaben aufgestellt.* Read before the *Medicinisch-Chirurgische Gesellschaft* in Berlin.

had been stretched into its present form by sexual intercourse and by irritation, and from the same causes made to acquire the great thickness and firmness it presented. Difficulty attended the division of the end of this sac, from the firmness of its texture, and the narrowness of the vagina; but this was effected by making a perforation in it, and then bringing it down as far as possible by the finger introduced into the incision. A rather copious hæmorrhage ensued. The absence of the uterus was now discovered: on introducing the finger beyond the opening in the sac, it encountered a soft, spongy, penetrable, substance, evidently a mass of cellular tissue. Some similar cases, which Professor Stein found by searching literary records, which we shall immediately notice, with a subsequent examination, confirmed him in his former conclusion. The wound united very well by the use of bandages, &c. and no serious accident ensued.

We shall first adduce the account given by Theden of a case precisely similiar in character to that of Professor Stein; and then a description of the appearances discovered, on dissection, in two bodies, by Engel and Schmucker.

Theden's case was that of a young woman who had never menstruated, although the orgasm had regularly been manifest. Marriage was advised, as a probable means for remedying this disorder; and it was accordingly resorted to. An obstruction of the vagina was then detected: but the husband had, before Theden was consulted on the case, distended the obstructing membrane into a sort of pouch of the size of a hen's egg: this was hanging without the external orifice of the vagina, and was removed by incision. On introducing the finger behind the pubes, a cavity was discovered, terminated by a mass of cellular texture, through which the pressure of the intestines was supposed to be discerned; but not the smallest evidence of the existence of a uterus could be discovered. The incised parts united very well, and no accident is said to have ensued.

Schmucker, in his *Vermischte Schriften,* speaks of a body that was brought to him, which had been found in a field, presenting, externally, the female characteristics: the breasts were remarkably prominent; but the genital parts were of small size, and the orifice of the vagina closed by a membrane, externally, apparently a continuation of the skin. The uterus and vagina were wanting; the place which the former should have occupied, was filled with a mass of loose cellular texture, in which the ovaries and fallopian tubes were situated.

Engel's patient was also a woman whose habits were not known: it appears that the body accidentally came under his observation. The breasts were remarkably full; the orifice of the genitals was closed; there was neither vagina nor uterus: the ovaries and fallopian tubes were found attached to the bladder.

The physiological indications those cases furnish had been already pretty well ascertained; but they serve to confirm the opinion that it is the ovaries in which the menstrual orgasm originates, and which, by their influence, give to the woman her characteristics in respect to form and manners. When the ovaries have been originally wanting, the hermaphoditical character of the body generally has been constantly present: Pott describes the woman whose ovaries he excised from the groins, (presenting in the usual way of hernia,) mistaking them for diseased glands, as having lost her feminine appearance; her breasts, which had been full, sunk away, she became more muscular, and altogether somewhat man-like.

The medical indications which the foregoing cases present are sufficiently obvious; and should not be forgotten. For, although instances of a preternatural membrane, called the hymen, closing the orifice of the vagina, are common, and such cases as that of Dr. Stein very rare, yet the latter may occur to any practitioner, and the termination of others may not be so fortunate. Those who read this, will, in future, on dividing the membrane alluded to, first make only such an opening as will admit a bougie; if examination with this proves satisfactory, the incision may be extended so as to admit a finger; and then the operation may be fully effected.

---

### *The History of a remarkable Case of Convulsive Affection.* By Dr. Schroeder, of Thuringen.*

We generally offer some remarks on the subjects selected as worthy the attention of our readers, but on this interesting and extraordinary case we can form no satisfactory opinion: those of Dr. Schroeder are in opposition to the generally-received doctrine of *gout;* and yet they are rendered feasible by many points in the history of the disease, as well as of those attending its cure. We shall confine ourselves to a precise translation of the original.

Amalia Jaeger, seven years of age, a well-grown, blooming, lively girl, had, when four years old, a moveable, firm, tumor appear on the back of the hand. Several times a-day she has experienced in it, without any evident cause, a darting sensation, which runs along the arm like an *aura epileptica,* extending to the neck and face. The tongue then becomes stiff and hard, and the jaw affected with clonic spasms, which last a quarter of an hour. While the spasms continue she remains sensible, hearing all that is spoken without being able to speak. Three weeks since, a similar swelling appeared in the right knee; which, however, was dispersed by fumigation with mastich, amber, and juniper-berries, which the mother used as domestic remedies. The child then suffered an indeterminately periodical pain in the foot; which, however, came on in the night, and went off with a copious sweating. She was in other respects healthy; had a good appetite, a clean tongue, a natural daily motion of her bowels: but her sleep was very disturbed with startings during the presence of the pain.

There was much difficulty in ascertaining the nature of this case. There was no hereditary disposition to spasmodic affections in the family; worms had never been particularly observed; and but a single instance of the passing of a few ascarides from the patient had been remarked. She had an eruption on the head in early infancy, but no signs of scrofula were evident. The only cause that could with any probability be assigned for it, was *gout.* Her father had suffered very severely from gout

---

* From the *Achter und Neunter Jahresbericht des Königl. Poliklinischen Instituts der Universität zu Berlin, von den Jahren* 1817 *und* 1818. Vom Prof. C. W. Hufeland. Journal der Practischen Heilkunde des Berlin. Junius, 1819.

for many years, and he had a son who died at the age of ten years, who had several times been confined to his bed by attacks of that disease. It seemed, then, probable that the disease was an imperfect form of gout, which was concentrated in the hand, and caused the spasmodic complaint, from the circumstances already noticed, and from the sweating always being followed by relief, and the tumor of the hand becoming smaller, and the spasms ceasing, when the knee became affected.

I therefore ordered, in the first instance, as a vermifuge, the *semen santonici*; and then, to correct the *dispositio arthritica*, the *resina guaiaci* and *extractum aconiti*, inwardly; and, as an outward application, an embrocation of *ol. petræ* and *terebinthinæ*, joined with the sulphur-bath; and, in case the digestion should be deranged by the medicines, that they should be changed for an infusion of valerian with bitters. No worms were evacuated; the swelling of the hand became much less; the pain in the right knee got more severe, and was accompanied by great general uneasiness, with heat and dryness of the skin, which terminated by a copious acid sweat. On this change in the character of the malady taking place, the spasmodic attacks disappeared; and, at length, the gouty affection also. The treatment was commenced on the 5th of June, and terminated on the 18th of July, when the disease was perfectly removed.

---

### *An Account of a Case in which Gastrotomy was performed.* By Dr. Cayroche, of Mendes.

[*Bulletin de la Faculté de Médecine de Paris*, No. viii. 1819.]

We shall give our readers an abstract, rather than a full account, of the original history of this case: omitting, however, only those circumstances not essentially important in its medical relations.

Madame S—, 24 years of age, the mother of several children, of a sanguineo-choleric temperament, and of a robust habit, experienced, the 14th September, 1818, a sensation of weight about the stomach and inclination to vomit. With the view of inducing vomiting, she introduced into the œsophagus a silver fork, which excited so forcible a contraction in that canal, that it escaped from her hand and passed into the stomach.

The fork remained in the stomach during three months, without causing any other inconvenience than a sense of weight in that organ. Her ordinary physician being consulted at the end of this time, discovered that the handle of the fork was situate at the distance of two fingers' breadth above and a little to the right side of the umbilicus; whilst the prongs appeared to be placed in that part of the stomach below the liver. Madame S— ate and digested her food as well as usual; and the physician hoped that in the course of time the fork might become displaced, and pass by the intestines.

Towards the fifth month the patient suffered violent vomiting after indigestion; and at this instant the fork changed its position. The handle now was to be felt about two fingers' breadth above the umbilicus, and at the distance of two inches from the linea alba on the left side; the other extremity appeared to be situate in the right hypochondre, a little on that side of the vertebral column. From this time the patient became a prey to pains more or less severe, and she was perishing in a sensible manner, although the appetite continued to be good, and the alvine and uterine evacuations regular. She in vain offered to submit to an operation, although its consequences might be dangerous: it was determined that the results of the natural efforts should be waited for.

A second attack of vomiting, which came on towards the end of the sixth month, was followed by tumefaction externally, which acquired in a few days considerable bulk. The tumor was of the size of a hen's egg, and very prominent, but without redness of the skin: it corresponded with one of the extremities of the fork, which could be made to perform oscillatory motions by pressure with the hands; these were attended with very severe pain. The patient now was obliged to keep her bed, as walking, or the erect position of the body, excited great uneasiness. A consultation of MM. Blauquet, Barbut, and Cayroche, was then called, and the operation of gastrotomy was thought advisable; but it was determined to obtain first the opinion of MM. Delpech and Fages, celebrated professors at the University of Montpellier. From the letter of these physicians, it appeared evident that they would themselves resort to the operation under similar circumstances; it was therefore determined that it should be performed by M. Cayroche.

On the 1st of May, 1819, at eight o'clock in the morning, the season being calm and the atmosphere a little clouded, M. Cayroche, attended by several distinguished persons, performed the operation in the following manner: The patient was placed on a mattress on a long table, and held in a fixed position by the assistants. The first incision was made in the skin with a convex bistoury, in the most prominent part of the tumor, from above downwards, and to the extent of two inches. The fibres of the left rectus muscle were then divided down to the peritoneum. Two small arteries were opened, but twisting them with pincers arrested the hæmorrhage. After being assured that the two surfaces of the peritoneum adhered, and having made the fork project by pressing on its posterior extremity, a third incision divided the paries of the stomach, and the fork was exposed. The prongs of it were so implanted in the substance of the stomach, that M. Cayroche was obliged to disengage them successively by dissection, in order to avoid laceration: this being terminated, the fork was readily removed. No hæmorrhage ensued from the wound of the stomach: only some wind, and a little gastric fluid mingled with frothy mucus, escaped. The wound was lightly dressed with a pledget of lint only. The fork was about eight inches and a half long (English measure), and at its greatest breadth it measured a little more than one inch: it weighed three ounces and fifty-eight grains; and it was covered with a hard blackish crust, which could not be removed without considerable friction.

In the afternoon eight ounces of blood were taken from the right arm, which presented the buffy coat; the coagulum floated in a large proportion of serum.

The 2d of May, the patient had passed the night in calmness, and had slept at different intervals; the morning passed without disturbance. Towards the evening traumatic fever was manifested. Fourteen ounces of blood were taken from the arm; it was less buffed than that of the preceding evening. The patient slept forty minutes after the bleeding. She experienced very intense thirst, which was appeased by giving her from time to time a few spoonfuls of decoction of mallows, slightly acidulated with lemon-juice: part of this drink, however, passed by the wound, accompanied by colic pains, which disappeared on the escape of some flatus. The thirst was then appeased by moistening the mouth with some slices of orange. The wound was fomented, and dressed with simple lint.

The 3d of May, the patient had been in a state of repose during the greater part of the preceding night. The wound was but slightly painful, and suppuration well established; the patient could move about a little without uneasiness; a glyster of broth was given in the morning, and another soon after noon. Towards the approach of night the patient experienced a little general disturbance, and some colic pains in the bowels; the pulse was accelerated, the face a little coloured, and the respiration and speech a little obstructed. Another bleeding was thought advisable; but, as the patient expressed extreme repugnance to undergo it, it was omitted. The abdomen was fomented with warm linen, and a state of calmness soon returned. From this time neither liquids nor flatus escaped from the wound.

The 4th of May,—a calm night, though less so than the preceding one. No pain in the stomach or bowels; no fever. A glyster of broth morning and evening; which, as well as the former ones, all passed with the urine. The patient drank a ptisan of prunes at liberty; and even swallowed some of the pulp of them, without the consent of M. Cayroche.

On the 5th of May she had a natural fæcal evacuation.

On the 6th the patient began to take solid aliment. She can turn about, and lie in all situations; she desires to get up; and the wound is closing rapidly.

On the 14th of May, Madame S— came down stairs to dine with her family. The granulations in the wound are nearly on a level with the skin.

20th of May, complete cicatrization of the wound. Madame S— enjoys the most perfect health: she experiences no pain nor thrilling sensation, either in walking or during digestion. The tumefaction noticed before the operation has quite disappeared.

---

***Observations respecting Cystoid Tumors, and the various Changes they undergo in the Human Body.*** [Extracted from a Memoir addressed to the *Athenée de Médecine* at Paris, by J. J. Rigal, First Surgeon to the Hospital of Gaillac, Department of Tarn, &c.*]

We adduce these observations as illustrations of the theory of Dr. Baron. The deep interest we feel respecting it, (participated, we hope, by our readers,) will, in future, incite us to collect with care every fact bearing any apparent relation to it. We omit, of course, the account of the operations performed by M. Rigal, in the cases we are about to relate, as well as some of his remarks on them, as not being intimately connected with the circumstance for which we ex-

* From the *Bibliotheque Medicale,* tome xliv.

pressly value his observations. M. Rigal, although evidently a well-informed surgeon in general, does not seem to be acquainted with any of the notions that have been entertained on the subject of his observations in England; for he prefaces his history of the cases with the queries—May an encysted tumor degenerate into, or become complicated with, cancer? Will the following cases serve at all to illustrate this question?

"*1st Case.*—Mrs. M—, of Lisle, 36 years of age, of a robust constitution, had for several years a moveable, hydatidical,* tumor, situate in the middle, anterior, and a little exterior, part of the right leg; from which, for a long time, she suffered no inconvenience. The tumor at length changed its character: it became hard, and increased in size, so that in the space of two years it occupied almost the whole extent of this extremity. The cutaneous veins were dilated, and became varicose; lancinating pains, at first occurring but rarely, began to be experienced; these pains increased, and became more frequent. The patient lost her sleep; the appetite became depraved, and the general health fell off from day to day. The centre of the tumor then became prominent; it opened, and a small quantity of sanious and fetid matter escaped: the edges of the ulcer which resulted from this became reversed, a black spot appeared in its centre, frequent hæmorrhage took place, the pains continued: every thing characterized a real cancer. On being consulted on the state of this woman by Dr. Compayre of Lisle, after having by a careful examination convinced myself that the tumor was in some degree moveable, although its base extended from an inch from the external ankle to an equal distance from the condyle of the tibia, I believed that I might propose an operation for its removal.' This was consented to by the patient. The following is M. Rigal's description of the tumor: "The tumor, which weighed two pounds and a half, was found to present, in some points, a substance of a lardaceous consistence; in others, it was cartilaginous; whilst in the centre we found a cyst, of the size of a large egg, and full half a line in thickness, which had not undergone any alteration, and which was filled with concrete albuminous matter."

"*2d Case.*—Twenty years since, Madame d'Aires, of Mailhoc, was operated on by me with entire success (for she now enjoys good health,) for a cancer of the breast, which had commenced in the same manner as the foregoing case. The tumor, examined after the operation, offered the same characters as that of the former patient. There was a cyst in the centre, of the size of a rose-bud, not having undergone any alteration, and filled with an analogous substance. Madame d'Aires was then forty years of age: her menses had been interrupted for about four months, which was also the case with Mrs. M.; but they became re-established in both after the cure."

"*3d Case.*—Miss Icher, of Lisle, about 40 years of age, had a soft hydatidical tumor on the fore part of the right knee, which occasioned not the least inconvenience; but it having for a year past consider-

---

* *Loupeuse* in the original: the cystoid tumor, which we term generally an hydatid, is signified.—Edit.

ably increased in size, and at the same time become hard and tuberculous, and causing lancinating pains, I was consulted: my advice was the operation."—" A cyst, filled with matter similar to suet, was remarked at its anterior part, not participating at all with that in the schirrous state; for I detached it with facility from the rest of the tumor."

It would be imprudent in us not to adduce the following remarks of M. Rigal on the nature and origin of those cysts. He says,

" The observations I have made will not permit me to admit, neither that those cysts are formed by the cellular tissue, as Louis thought, nor the similitude which Bichat established between them and the serous membranes. The latter are thin and transparent, penetrated with blood-vessels, nerves, and lymphatics: these cysts, on the contrary, are deprived of them; they are opaque, and often of more than a line in thickness. If they were formed by the cellular tissue, they would not be detached from it with so little difficulty as they are. These cysts are, like the crystalline, isolated in the centre of our organs and of the different parts of the body, without any vessel terminating them; they are attached to the parts in the midst of which they are situated only by a sort of gluten or filament, I know not of what nature, but they adhere but very feebly."—" Perhaps these cysts may owe their formation to effused coagulable lymph, which, by the compression exerted on them in so many ways by the surrounding parts, receive the form more or less round that they always possess. If it be asked how these cysts are nourished, I would reply, that we can say nothing positive on this subject; but that their filamentous and spongy nature render them adapted to absorb, and to become penetrated by, the fluids which are continually exhaled by the surrounding parts in contact with them, and with which they are in immediate relation. But do not let us carry these conjectures further, lest the cloud of hypotheses should obscure well-observed facts."

---

We have promised from time to time to offer to our readers an exposition of the medical doctrine prevalent in Italy at the existing period, and of the progress of the revolution that has recently taken place; but an inability to complete our sources for literary information, obliges us still to defer it to some future occasion. In the mean time, however, this interesting subject shall not be wholly neglected: in the next Number we shall give an analysis of the work of Rasori, which comprises the elements of the new doctrine; and this will be followed by those of the productions of the other authors who have adopted the same principles, and applied them to febrile diseases in general; the most important part of the subject will thus be embraced. At present, from the little space left in this Number, we shall only produce the following observations, as interesting pathological facts; and here, as well as on future occasions, we shall endeavour to elucidate any expressions relating to opinions not generally understood, by such remarks as our personal observations and literary acquirements may enable us to offer.

*Some Pathological Observations;* by Signor Dottore FRANCESCO QUAGLIA DI ALESSANDRIA.

A girl, 17 years of age, of a sanguine and very excitable temperament, was attacked, after amenorrhœa of six months' duration, with an acute pain in the left side of the chest, difficulty of respiration, cough, ardent synocha, a sense of audible tremor internally in the part in pain, and energetic vibration of the large arteries of the chest. Notwithstanding moderate bleeding,* and the use of the most powerful contra-stimulants,† as digitalis, distilled laurel-water,‡ the extracts of hyosciamus and of belladonna, nux vomica, prussic acid, and antimonial preparations,§ the disease pertinaciously continued, assumed a chronic form, and the aspect, indeed, of a thoracic angina. The use of general tepid baths, of sarsaparilla, and of mercurial frictions to the situation of the pain, were then also resorted to, but without any advantage; since, although that mode of treatment was continued during fourteen months, the disease was not yet conquered.

In this state the patient exposed herself to the impression of cold air; and, with great agitation, and feelings of great alarm, she had an access of convulsive asthma, whilst, after two similar paroxysms in the two following days, she was successively affected with trismus, tetanus, opisthotonos, emprosthotonos, pleurosthotonos, and various irregular convulsive attacks, causing her sometimes to dance, sometimes to leap or to walk about, sometimes to clamber up a wall, and sometimes to agitate and contort the limbs in various ways. The centre of these strange affections appeared to be the dorsal portion of the spinal marrow, (as my distinguished tutor BORDA, who was called to visit the patient, judiciously remarked,) since all the course of the dorsal vertebræ was painful, and a little swelled. The principal nerves of the left arm were become so sensible, that, on feeling the pulse, or on touching the hollow of the axilla, the point of the shoulder, and the scapula, the patient suddenly drew away the arm, complaining of severe pain, which extended from the point pressed on to the spinal column; and was seized with convulsions over the whole of the body. This did not happen on touching the right arm. We opened a vein, applied leeches along the dorsal vertebræ; and exhibited the extracts of hyosciamus and belladonna, assafœtida, the sulphate of zinc, the wild valerian, general tepid baths; and had frictions, made with equal

---

* The abstraction of about eight or ten ounces, three or four times repeated, is, in Italy, in such a patient, considered as moderate blood-letting.—EDIT.

† For an explanation of this expression, see our review of Dr. Chapman's Discourses on Therapeutics.—EDIT.

‡ The principal medicinal qualities of this depend on the prussic acid it contains; and, as the strength of the latter preparation is so variable, even when made with considerable care, the laurel-water is, perhaps, the best mode of exhibiting that powerful medicine. Since the doctrine of RASORI has become prevalent, it has been a remedy commonly employed.—EDIT.

§ The tartar emetic is generally employed, and it is given in large doses, (for instance, from half a grain to a grain,) frequently repeated, as every two, three, or four hours: a considerable quantity will be thus borne without vomiting being excited after the first or second occurrence of that action.—EDIT.]

parts of oil of henbane-seeds and mercurial ointment, along the whole painful part of the spine. We, by this treatment, conquered the disease in the course of two months. But, from time to time, from some slight or unknown cause, she was affected with convulsions; and, at length, by some new exciting causes, with severe cough, great difficulty of respiration, and fever. I bled her four times, and gave her distilled laurel-water; and thus in a few days not only removed the new affection of the chest, and so perfectly extinguished all the remains of convulsions, that for a year since she has not experienced them.

---

*The History of a Case of Disease of the Lungs.* Related by a Disciple of the Clinical Medical School of Bologna.*

About the commencement of the year, the student S. G. born of healthy parents, and of a moderately robust temperament, after some gymnastic exercises, in which he engaged to an immoderate degree in early life, was suddenly affected with hæmoptysis; and many were the pounds of blood that he spat from his lungs. In the treatment of his disease, the most prompt contra-stimulant remedies were resorted to; bleeding was frequently repeated; with the external and internal use of ice, sulphuric acid, ipecacuanha, &c.; and with such constancy and courage, that he might consider it certain that it was by that alone that he had escaped from the danger he had undergone. Forty days after the cure of the former affection, he had a relapse, which was also removed by the same means. Although he afterwards took great care of his health, he could not escape a catarrh on the occurrence of the cold of winter. He returned again to this Clinical Institution, where he had formerly been rescued from death, but it was in vain: the use of antimonials, of the lauro-cerasus, ipecacuanha, lichen, poligala, &c. produced no benefit. The cough continued; became more obstinate; some febrile action came on at the turn of the evening, and he became manifestly emaciated in person. From these symptoms, and from the inefficacy of the medicines employed, which were sufficient evidences for the supposition of organic lesion, there appeared reason to believe that phthisis, so frequently the consequence of hæmoptysis, now existed. But, although the symptoms led to this afflicting diagnosis, there were no new indications for the mode of treatment: the continuance of the method already employed was only insisted on.

Whoever will recal to mind the sentiments of the best practitioners, will find them all agree in the opinion that phthisis is a low and chronic form of inflammation; and Morton, in the cure of it, recommends bleeding, which, "*ad flammam hecticam latescentem in sanguine vel extinguendam magis conducit;*" and he expressly asserts, that it happens that "*multi a peripneumonia, catarrhis communibus, aliisque ejusmodi morbis in fatales phthises præcipites ruunt, eo quod ex incuria medici, vel etiam ægri et adstantium amicorum metu sanguis non satis tempestive vel frequenter vel copiose per venæsectionem fuerit detractus.*"

* The doctrine professed there is that of Rasori, developed and illustrated by Professor Tommassini, whose eminent talents have given to it a character that has insured its success in Italy.—Edit.

With bleeding he desires to be joined purgatives, mucilages, diaphoretics, &c.; amongst which, he recommends that "*cæteris præferantur ea quæ minimum caloris et acrimoniæ sanguini impertiant.*" Stoll, too, in the cure of this disease, says, that "*venæ sectiones morbo et viribus ægri respondentes, decocta herbarum et radicum emollentium, emulsa et victus mere vegetabilis omnibus aliis remediis palmum preripiunt.*" And Morgagni, in his venerable treatise *De Sedibus et Causis Morborum*, which has so much contributed to aggrandize Italian medicine; and Portal, Lieutaud, Bonetus, and Bartholinus, and many others of similar character, all confess that they have witnessed in those who have died of that disease, the lungs inflamed, with suppuration, ulceration, &c. but never to have found an opposite condition. From these considerations, we exhibited the extract of aconite, and other species of contra-stimulants, in such doses as could be borne by the system generally, which was already gradually verging to a state the opposite of that of the affected viscera; and we permitted the patient to take such food as, without aggravating the disease, might afford to him light and necessary nutriment. But all these means were used in vain; it already acquired the state which necessarily ends in destruction. It was very afflicting to see this unhappy fellow, a short time since in vigorous health, become so soon reduced, and so early in life fall into extreme languor; his countenance showing the paleness of death; his body cold; his skin of a waxen colour; his pulse almost extinct, though showing very slight signs of febrile action. Nothing indicated inflammation in any part: the only evident signs, we may indeed say, of disease, were the very gentle progress of decline, the languid voice, and great difficulty of respiration. But, what opposite appearances to such langour of action presented themselves on dissection of the body! The lungs showed the characters of violent inflammation; the whole of their surface and the most intimate parts of their parenchymatous structure, was of a red colour, as if injected with minium; and they adhered to the sides of the chest and to the diaphragm, by recent and still fresh bands and vegetations. A person who was ignorant of the state in which the patient had been, would have necessarily believed those organs to have been those of a robust athletic man, who had died in the early stage of a most acute attack of pneumonia. I believe that, by reasoning on those circumstances, two conclusions may be drawn, which accord so well with the maxims of the new doctrine: that, even in the midst of the most desolating privation of physiological vigour, partial phlogosis may arise, be maintained, and increase until it destroys the structure of the organ it affects; and that it is wrong to argue that all diagnostic judgment should be formed from the external appearances; for what has been described shows clearly that the greatest decline of physiological power of the general system, and a most violent degree of local inflammation internally, may be combined.

